# LINGO-1 regulates Wnt5a signaling during neural stem and progenitor cell differentiation by modulating miR-15b-3p levels

**DOI:** 10.1186/s13287-021-02452-0

**Published:** 2021-06-29

**Authors:** Chen-Guang Zhao, Jie Qin, Jia Li, Shan Jiang, Fen Ju, Wei Sun, Zhen Ren, Yu-Qiang Ji, Rui Wang, Xiao-Long Sun, Xiang Mou, Hua Yuan

**Affiliations:** 1 Department of Rehabilitation Medicine, Xijing Hospital, Fourth Military Medical University, Xi’an, China; 2 Department of Orthopedics, The Second Affiliated Hospital of Xi’an Jiaotong University, Xi’an, China; 3 Department of Medicine and Health, University Bretagne Occidentale, Brest, France; 4 Department of Rehabilitation Medicine, China-Japan Friendship Hospital, Beijing, China; 5 Department of Ultrasound, Xijing Hospital, Fourth Military Medical University, Xi’an, China; 6 Department of Central Laboratory, The First Hospital of Xi’an, Xi’an, China

**Keywords:** LINGO-1, Neural stem and progenitor cells, Spinal cord injury, Differentiation, Wnt5a, miR-15b-3p

## Abstract

**Background:**

Manipulation of neural stem and progenitor cells (NSPCs) is critical for the successful treatment of spinal cord injury (SCI) by NSPC transplantation, since their differentiation into neurons and oligodendrocytes can be inhibited by factors present in inflamed myelin. In this study, we examined the effects of LINGO-1 on spinal cord-derived NSPC (sp-NSPC) differentiation, the underlying mechanisms of action, and the functional recovery of mice after transplantation of manipulated cells.

**Methods:**

sp-NSPCs were harvested from female adult C57/BL6 mice after SCI induced with an NYU impactor. These cells were infected with lentiviral vectors containing LINGO-1 shRNA sequence or a scrambled control and transplanted into SCI mice. Tuj-1- and GFAP-positive cells were assessed by immunofluorescence staining. Wnt5a, p-JNK, JNK, and β-catenin expression was determined by Western blot and RT-qPCR. miRNAs were sequenced to detect changes in miRNA expression. Motor function was evaluated 0–35 days post-surgery by means of the Basso Mouse Scale (BMS) and by the rotarod performance test.

**Results:**

We discovered that LINGO-1 shRNA increased neuronal differentiation of sp-NSPCs while decreasing astrocyte differentiation. These effects were accompanied by elevated Wnt5a protein expression, but unexpectedly, no changes in Wnt5a mRNA levels. miRNA-sequence analysis demonstrated that miR-15b-3p was a downstream mediator of LINGO-1 which suppressed Wnt5a expression. Transplantation of LINGO-1 shRNA-treated sp-NSPCs into SCI mice promoted neural differentiation, wound compaction, and motor function recovery.

**Conclusions:**

LINGO-1 shRNA promotes neural differentiation of sp-NSPCs and Wnt5a expression, probably by downregulating miR-15b-3p. Transplantation of LINGO-1 shRNA-treated NSPCs promotes recovery of motor function after SCI, highlighting its potential as a target for SCI treatment.

## Background

Spinal cord injury (SCI) is a serious central nervous system condition which can result in permanent functional impairment [1]. Promoting the differentiation of exogenous and endogenous neural stem and progenitor cells (NSPCs) into neurons is one of the most promising approaches for the treatment of SCI. However, there are numerous inflammatory factors which can affect this process of differentiation, such as myelin-associated inhibitory factors (MAIFs), which trigger cellular responses by binding to the NgR/LINGO-1/p75 (or TROY) receptor complex [2].

LINGO-1 is a central nervous system-specific 614 amino acid protein encoded by a gene located on chromosome 15 (15q24.3) [3]. It has many biological functions, including negative regulation of axon regeneration, neuron survival, and oligodendrocyte differentiation and remyelination. Studies have revealed that inhibition of LINGO-1 can promote axonal integrity in autoimmune encephalomyelitis [4], enhance the function of dopaminergic neurons in Parkinson’s disease models [5], and improve remyelination in multiple sclerosis [6]. Therefore, LINGO-1 is a potential therapeutic target for the treatment of neurological diseases. It has also been reported that LINGO-1 is a negative regulator of neural differentiation of NSPCs [7], so downregulation of LINGO-1 could promote differentiation of NSPCs into neurons, but the underlying mechanisms remain elusive.

MicroRNA (miRNA) are small, highly conserved, non-coding RNA molecules involved in the post-transcriptional regulation of gene expression [8]. Mature miRNAs are produced from precursor miRNAs (pre-miRNAs) by a Dicer-containing enzyme complex, and pre-miRNAs are processed from primary miRNAs (pri-miRNAs) by Drosha. miRNAs can target mRNAs, resulting in translational repression. Many miRNAs are altered following SCI [9]. However, the relationship between LINGO-1 expression and changes in miRNAs is unclear.

Through RNA sequencing (RNA-seq), we found that LINGO-1 is an upstream regulator of miR-15b-3p and that it relies on this mechanism to regulate the Wnt signaling pathway. Downregulation of LINGO-1 in spinal cord-derived NSPCs (sp-NSPCs) promoted Wnt5a expression and neural differentiation. Overall, our study revealed a LINGO-1/miR-15b-3p/Wnt5a pathway which can modulate spinal cord-derived NSPC differentiation. Its elucidation may contribute to the treatment of spinal cord injury.

## Methods

### SCI and spinal cord-derived NSPC culture

All animal experiments in this study were carried out in accordance with the National Institute of Health guide for the care and use of Laboratory animals (NIH Publications No. 80-23) revised 1996, approved by the Medical Ethics Committee at Xijing Hospital of the Fourth Military Medical University, and all efforts were made to minimize animal suffering and reduce animals used.

**Fig. 1:**
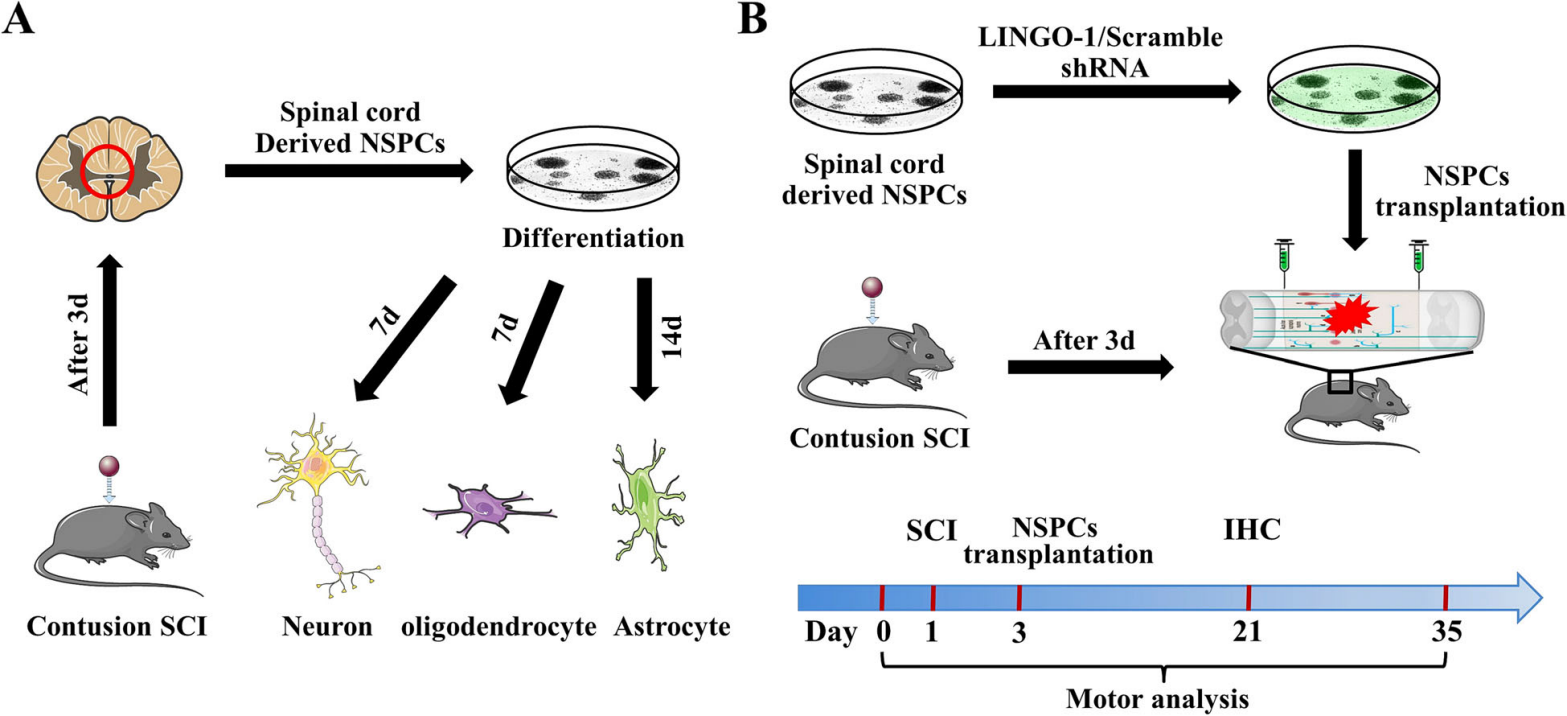
Schematic illustration of sp-NSPC culture, differentiation, and experimental design. **A** sp-NSPCs, harvested from injured spinal cords, differentiated into neurons, astrocytes, and oligodendrocytes. **B** Transplantation of LINGO-1 shRNA-treated sp-NSPCs and timeline for motor function analysis

Briefly, three 6-week-old female adult C57/BL6 mice were anesthetized with 5% isoflurane and subjected to laminectomy at the T9–10 vertebral level. Contusion SCI was induced with an NYU impactor by dropping a 5-g rod 6.25 mm above the spinal cord. After skin closure, mice were placed on a warming pad until they were completely awake. Manual bladder emptying was done twice a day for 3 days. The mice with SCI were anesthetized again, and their T6–T12 spinal cords were isolated, placed in 10-cm dishes containing cold PBS, dissociated, and filtered through a 70-μm strainer (BD Falcon). The cells were plated at a density of 1 × 10^6^ cells per mL in NSPC growth media (Dulbecco’s modified Eagle medium, DMEM/F12, B-27, N-2, Gibco) and passaged every 7 days (Fig. 1A).

### RNA interference

The LINGO-1 shRNA sequence and lentiviral vector carrying the green fluorescent protein (GFP) were designed and constructed by Genechem (Shanghai, China). The oligonucleotides were ligated into Age I- and EcoR I-digested GV248-RNAi. The LINGO-1 shRNA sequence is 5′-CCGGCAGTGAGAACAAGATCGTCAT CTCGA GATGACGATCTTGTTCTCACTGTTTTTG-3′. The scramble (negative control) shRNA sequence is 5′-CCGGTTCTCCGAACGTGTCACGTTTCAAGAGAACGT GACACGTTCGGAGAA-3′. sp-NSPCs were infected with the lentiviruses at a MOI of 20. The infection efficiency was verified by GFP expression using fluorescent microscopy.

### Differentiation of sp-NSPCs

For induction of cell differentiation, we followed the Gibco Neurobiology Protocol Handbook, as previously described [10]. sp-NSPCs were allowed to adhere to cover slips coated with poly-l-lysine (0.01%, P4707, Sigma) and laminin (10 μg/mL, L2020, Sigma) in NSPC growth medium for 1 day. Then, the medium was changed to a differentiation medium, as recommended by the Gibco Neurobiology Protocol Handbook. Neurons and oligodendrocytes were differentiated for 7 days, while astrocytes were differentiated for 14 days.

### Establishment of the SCI animal model and NSPC transplantation

Twenty-four 8-week-old female C57BL/6 mice were used in the animal experiments. Mice were randomly divided into two groups: (1) control group (SCI + control NSPC transplantation) and (2) LINGO-1 shRNA group (SCI + LINGO-1 shRNA NSPC transplantation). After anesthesia with 5% isoflurane, mice were subjected to laminectomy at the T9–10 vertebral level. Contusion SCI was induced with an NYU impactor by dropping a 5-g rod 6.25 mm above the spinal cord to produce a mild injury. Manual bladder emptying was done twice a day until urinary function was restored. Three days after SCI, the mice were anesthetized once more and their spinal cords were re-exposed. A total of 1 μL of LINGO-1 shRNA or control sp-NSPCs (1 × 10^5^ cells/1 μL PBS) were microinjected 2 mm rostral and caudal to the lesion epicenter. After skin closure, mice were placed on a warming pad until they were totally awake. Animals were maintained for functional assessment before being sacrificed (Fig. 1B).

### Quantitative real-time PCR

Total RNA was extracted using TRIzol reagent (Invitrogen) and cDNA was reverse transcribed using PrimeScript RT Reagent Kit with gDNA Eraser (TaKaRa). qRT-PCR was performed using TB Green Premix Ex Taq II (TaKaRa). mRNA levels were expressed as fold change after normalization to β-actin levels (used as internal control), and data were analyzed by means of the 2^*−△△Ct*^ method. The primer pairs had the following sequences: LINGO-1: CTTTCCCCTTCGACATCAAGAC (forward), CAGCAGCACCAGGCAGAA (reverse); Wnt5a: CAACTGGCAGGACTTTCTCAA (forward), CATCTCC GATGCCGGAACT (reverse).

### Immunofluorescence staining and cell counting

For immunohistochemistry, mice were perfused transcardially with 4% paraformaldehyde (PFA). Tissues were then sectioned, mounted onto slides, and stored at −80 °C until use. For cell culture staining, cells (NSPC spheres, adherent NSPCs, differentiated neural or glial cells) were fixed with 4% paraformaldehyde and mounted onto slides after staining. All samples were blocked for 1 h with 10% normal goat serum in PBS supplemented with 0.3 % Triton X-100. Samples were incubated with primary antibodies overnight at 4 °C. After washing with PBS, samples were incubated with secondary antibodies (1:500, Abcam) and DAPI (1:1000, Abcam) and analyzed by fluorescence microscopy (Zeiss Axio Vert. A1). The following primary antibodies were used: anti-nestin (NSPC marker, 1:200, Chemicon), anti-Tuj1 (neuronal marker, 1:200, Millipore), anti-DCX (neuronal marker, 1:100, CST), anti-GFAP (astrocyte marker, 1:400, Dako), anti-O4 (oligodendrocyte marker, 1:50, Chemicon), anti-CSPG (1:200, Abcam), and anti-LINGO-1 (1:200, Abcam). The percentage of positive cells was calculated based on three biological replicates.

### Western blotting

After washing with ice-cold PBS, cells were lysed with RIPA lysis buffer (Thermo Fisher) containing protease inhibitor (Roche). The protein concentration in the lysates was measured using a NanoDrop spectrophotometer (ND-1000, Thermo Fisher), loading 20 μg of sample for each analysis. After separation in a 10% SDS-PAGE gel, proteins were transferred to polyvinylidene fluoride (PVDF) membranes, which were blocked with 5% skim milk in Tris-buffered saline plus Tween (TBST). The blots were incubated at 4 °C overnight with the following primary antibodies: anti-LINGO-1 (1:500, Abcam), anti-Wnt5a (1:250, R&D Systems), anti-JNK (1:800, Santa Cruz), anti-p-JNK (1:800, Santa Cruz), anti-β-catenin (1:1000, Cell Signaling Technology), and anti-β-actin (1:2000, Cell Signaling Technology). After washing with TBST, the membranes were incubated for 1 h with the appropriate secondary antibody (1:2000, Abcam). The developed X-ray films were scanned and images were analyzed quantitatively with ImageJ software (version 1.45, NIH, USA). Relative protein levels were expressed as fold change after normalization to β-actin.

### miRNA-sequence analysis

LINGO-1 shRNA and control NSPCs were harvested and total RNA was extracted using TRIzol reagent (Invitrogen), according to the manufacturer’s instructions. Sequencing was performed by the Novogene Company (Beijing, China). Sequencing libraries were generated using NEBNextR Multiplex Small RNA Library Prep Set for Illumina RR (NEB, USA) and RNA quality was assessed using the Agilent Bioanalyzer 2100 system. The libraries were sequenced using an Illumina HiSeq 2500 platform. The miRNA error rate for each sample was < 0.01%. Clean reads with a length of 21–22 nt were screened as miRNA and mapped to the reference transcriptome sequence with bowtie.

### Luciferase assay

On day 1, HEK293 cells were plated in a 6-well plate at a density of 0.5 × 10^6^ cells per well. The following day, the cells were transfected with 250 ng of pmirGLO Dual-Luciferase miRNA Target Expression Vector (E1330 from Promega) containing Wnt5a 3′-UTR sequence, together with miR-15b-3p mimics or miRNA mimic control (Genepharma) at a final concentration of 50 nM. LNA inhibitors (Exiqon) were used at a concentration of 20 nM. Experiments were performed in triplicate. Luciferase activity was measured 72 h post-transfection using the Dual-Luciferase Reporter Assay System (E1960 from Promega) according to the manufacturer’s protocol. *Renilla* luciferase activity was normalized with respect to firefly luciferase activity and the percentage inhibition was calculated.

### Analysis of motor function

Mice were acclimated to the testing room and apparatus for 1 h before behavioral testing. Two well-trained observers, blinded to the genotypes or treatments performed, independently assessed the results, and the consensus score was analyzed.

Hindlimb movements were evaluated using the 9-point Basso Mouse Scale (BMS), with scores ranging from 0 (complete paralysis) to 9 (normal mobility). The 11-point BMS subscore was also assessed. Locomotor activity was observed by placing the mice at the center of a plastic enclosure with a smooth, non-slippery floor. Mice showing a difference of more than 2 scoring points between their left and right hindlimbs were excluded.

For the rotarod performance test, an accelerating rotarod was used to measure motor capability by accelerating the rod from 0 to 40 r.p.m. Each mouse was tested three times with a minimum interval of 30 min between trials.

### Statistical analysis

Data were expressed as the mean ± SD and analyzed using SPSS software (version 20.0 IBM, Chicago, IL). Significant differences between two groups were calculated by means of the Student t-test. BMS and BMS subscores at different timepoints were analyzed by means of repeated measures analysis of variance (rmANOVA). A post hoc analysis with Bonferroni’s adjustment was conducted for further multiple comparisons. The level of significance for all comparisons was set at *P* < 0.05.

## Results

### Spinal cord-derived NSPCs show multi-directional differentiation potential

**Fig. 2:**
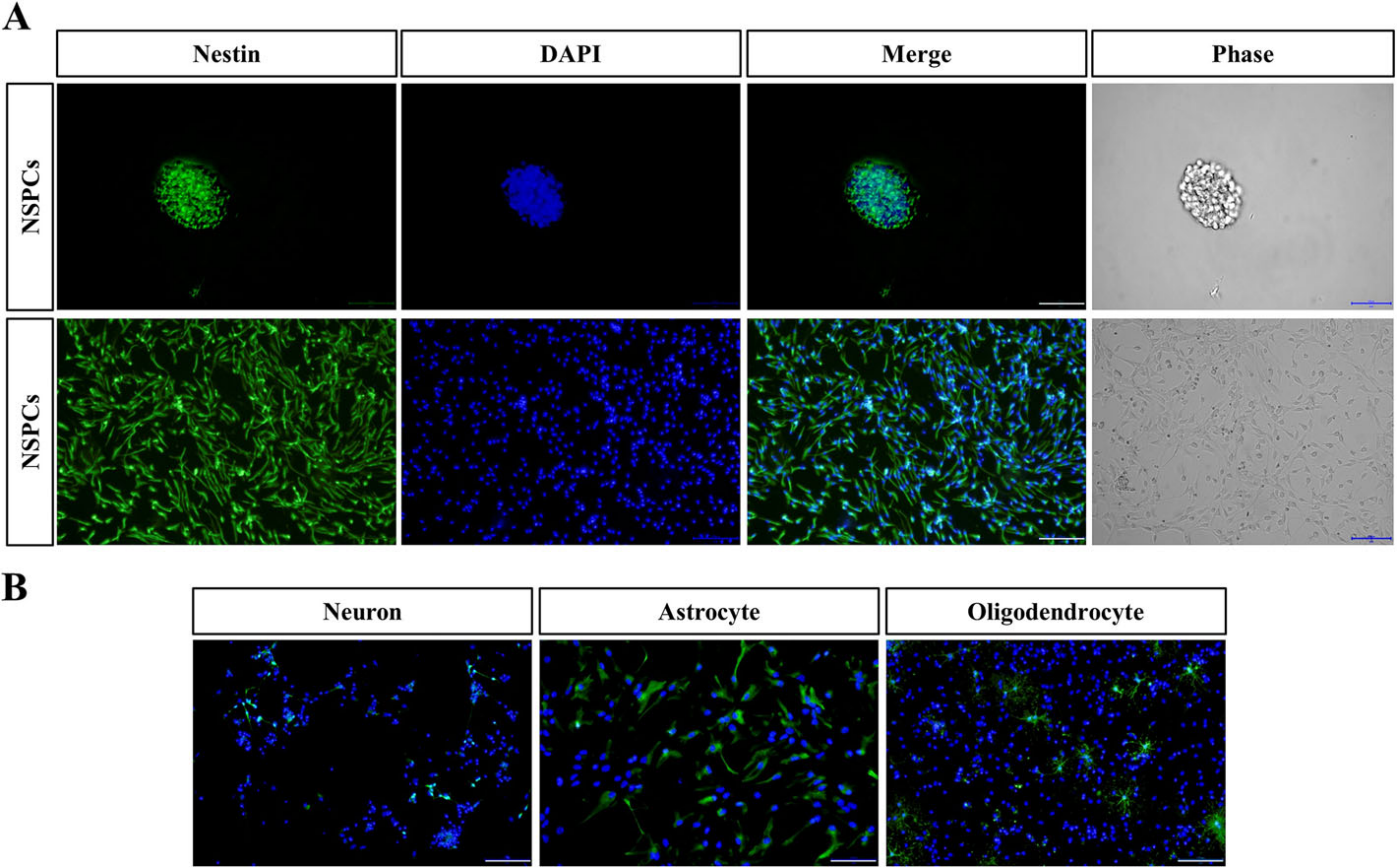
Primary adult mouse sp-NSPCs cultured under different conditions show divergent differentiation. **A** Neurosphere formation in suspension cultures (bar = 50 μm) and sp-NSPCs in adherent cultures (bar = 100 μm). Cells were labeled with nestin-specific antibodies. **B** Immunofluorescent staining shows sp-NSPC differentiation into Tuj-1-positive neurons, GFAP-positive astrocytes, and O4-positive oligodendrocytes. Nuclei were stained with DAPI (blue) (bar = 100 μm). The analysis was calculated based on three biological replicates

NSPCs derived from adult mouse spinal cords were cultured for subsequent experiments. A total of 97.9 ± 1.1% of the NSPCs expressed the neural stem cell marker nestin (Fig. 2A). Neurospheres formed 3 days after isolation (Fig. 2A, first line). After being seeded on coated cover slips, sp-NSPC morphology changed to unipolar or bipolar cells with short processes (Fig. 2A, second line), and after addition of distinct differentiation media and culture for a number of days (as described in the “Methods” section), 10.1 ± 1.7% of the cells were Tuj-1 positive, 61.7 ± 3.1% cells were GFAP positive, and 9.4 ± 1.2% of the cells were O4 positive (Fig. 2B).

### LINGO-1 is expressed by sp-NSPCs, neurons, and oligodendrocytes, but not by astrocytes, and its expression increases over time

**Fig. 3:**
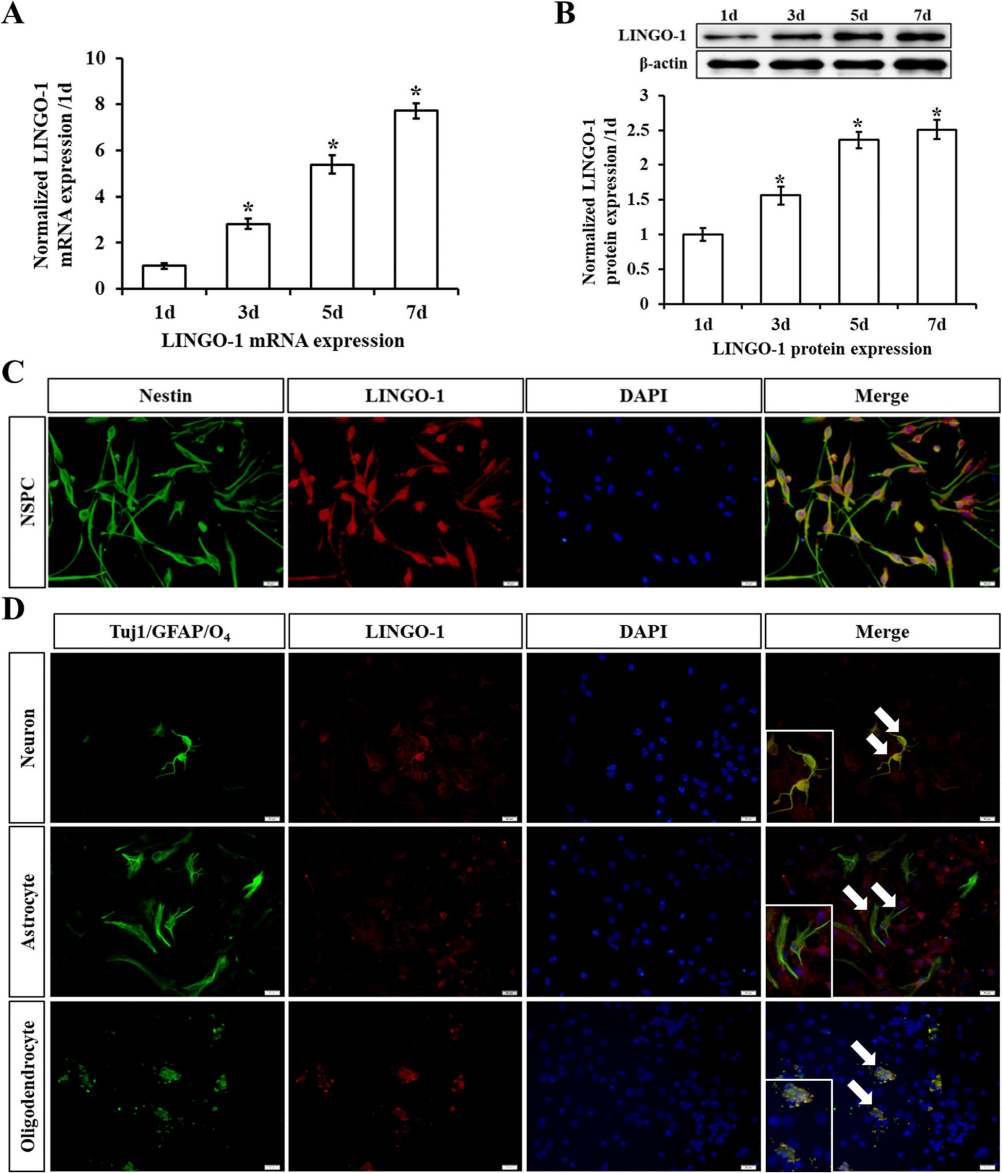
Expression of LINGO-1 by sp-NSPCs and differentiated cells. **A** LINGO-1 mRNA and **B** LINGO-1 protein expression levels increased significantly during differentiation, as revealed by quantitative real-time PCR and Western blot analyses. Double immunostaining shows **C** LINGO-1/Nestin-positive sp-NSPCs cultured in NSPC growth media and differentiated into **D** LINGO1/Tuj-1-positive neurons, LINGO-1/GFAP-positive astrocytes, and LINGO-1/O4-positive oligodendrocytes (arrows) when the medium was changed to the differentiation medium. Enlarged areas show cells at higher magnification. Nuclei were stained with DAPI (blue) (bar = 20 μm), ^*^P < 0.05. Results are expressed as the mean ± SD. All of the analysis was calculated based on three biological replicates

An analysis of the expression of LINGO-1 over time during sp-NSPC differentiation was performed by qRT-PCR and Western blot. Levels of LINGO-1 mRNA increased from the first day, and its expression by days 3, 5, and 7 was significantly higher than in day 1 (*P* < 0.05) (Fig. 3A). Peak mRNA expression (7.73-fold compared with day 1) was reached on day 7. LINGO-1 protein expression levels showed a similar trend (*P* < 0.05), although there were no significant differences in protein expression levels between days 5 and 7 (*P* > 0.05). Quantification of LINGO-1 protein levels showed a 2.36-fold and 2.51-fold increase by days 5 and 7 of differentiation, respectively, when compared with day 1 (Fig. 3B).

Next, we investigated LINGO-1 expression by different cells during differentiation through double immunofluorescence staining. We found that 94.5 ± 1.5% of cells expressed nestin at day 7 and 100% of the nestin-positive NSPCs expressed LINGO-1 (Fig. 3C). Differentiated cultures were stained with antibodies against LINGO-1 and Tuj-1 (neurons), GFAP (astrocytes), or O4 (oligodendrocytes) after 7 days or 14 days. Consistent with previous reports, our results showed that 100% of neurons and oligodendrocytes expressed LINGO-1, but astrocytes did not (Fig. 3D).

### LINGO-1 shRNA increased neuron differentiation while decreasing astrocyte differentiation

**Fig. 4:**
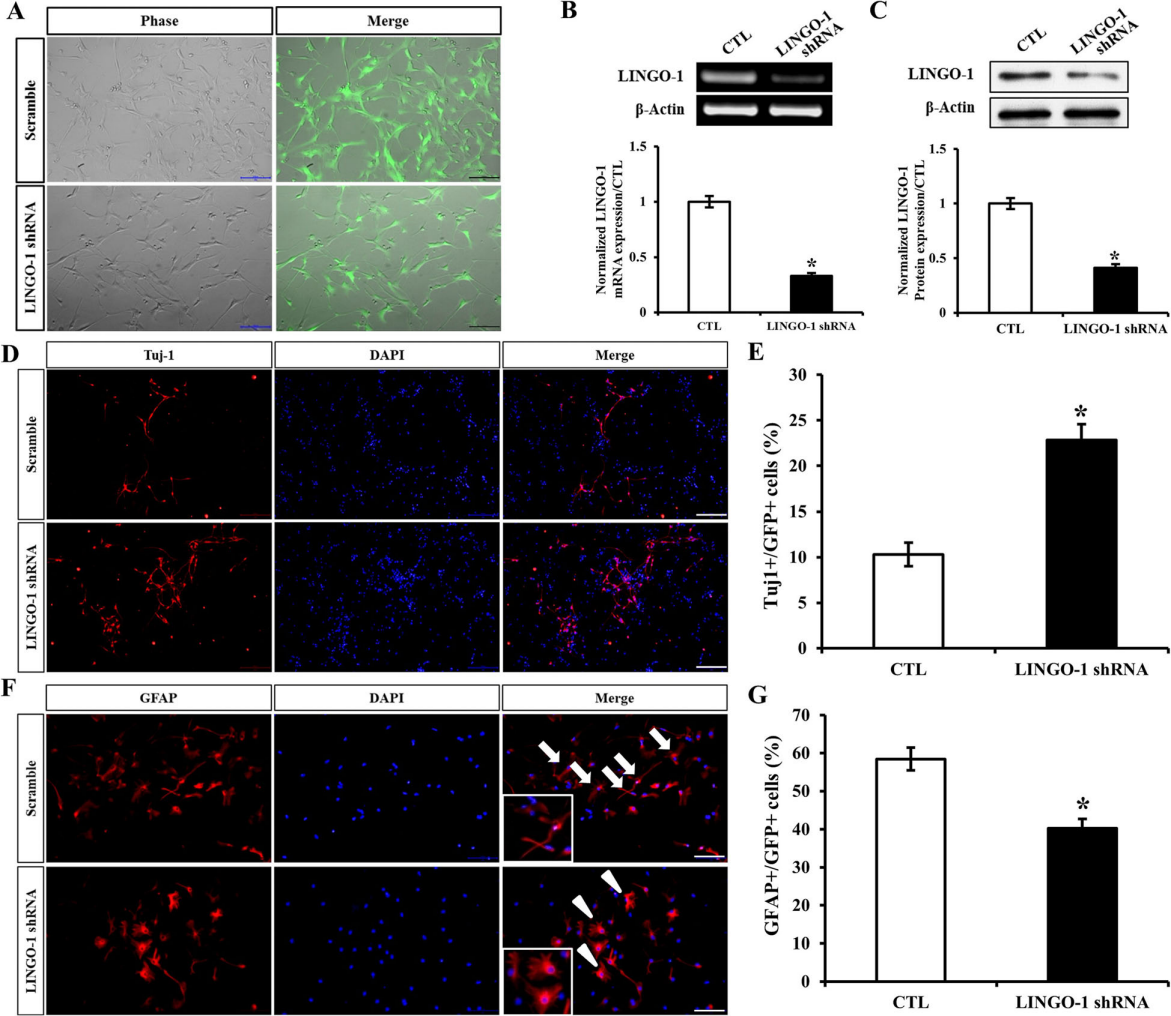
Effects of LINGO-1 shRNA on sp-NSPC differentiation. **A** sp-NSPCs were transfected with GFP-expressing LINGO-1shRNA lentiviral vectors. **B** LINGO-1 mRNA and **C** LINGO-1 protein expression levels decreased significantly. Immunofluorescence staining and quantification demonstrated that **D**, **E** Tuj1+/GFP+ neurons increased significantly, while **F**, **G** GFAP+/GFP+ astrocytes decreased in LINGO-1 shRNA-treated sp-NSPCs. Enlarged areas show that astrocytes in the LINGO-1shRNA group appeared more mature (arrowheads) than those in the control group (arrows) (bar = 100 μm), ^*^P < 0.05. Results are expressed as the mean ± SD. All of the analysis was calculated based on three biological replicates

We tested the effects of LINGO-1 RNA interference on cell differentiation. sp-NSPCs were incubated with GFP-expressing LINGO-1shRNA lentiviral vectors at a MOI = 20, and results showed that the transduction efficiency was over 90% (Fig. 4A). LINGO-1 mRNA levels were reduced to 24.5% of the control group (Fig. 4B) and LINGO-1 protein levels were reduced to 32.1% of the control group (Fig. 4C).

To elucidate the effects of LINGO-1 on the differentiation of sp-NSPCs, we performed immunofluorescence assays after culturing the cells in distinct differentiation culture media for 7 days (for neurons) or 14 days (for astrocytes). Our results demonstrated that LINGO-1 mRNA interference had a profound effect on sp-NSPC differentiation. For example, Tuj-1-positive cells in the LINGO-1 shRNA group were significantly more abundant than in the control group (Fig. 4D). Specifically, the percentage of Tuj-1-positive cells in the LINGO-1 shRNA group (21.7 ± 1.7%) was more than 2-fold higher than that in the control group (9.8 ± 1.3%) (*P* < 0.05) (Fig. 4E). In contrast to neural differentiation, the percentage of GFAP-positive cells in the LINGO-1 shRNA group (38.3 ± 2.3%) was much lower than that in the control group (56.1 ± 2.9%) (*P* < 0.05) (Fig. 4F, G).

Interestingly, when we identified astrocytes by staining them with GFAP-specific antibodies, we found that their morphology in the two groups was not the same. Astrocytes in the LINGO-1 shRNA group exhibited a more mature, ramified morphology, characterized by their star-like appearance (Fig. 4F, arrowheads and enlarged area). On the contrary, astrocytes in the control group showed an immature morphology, with a round phenotype and only short processes (Fig. 4F, arrows and enlarged area). Our results showed that downregulation of LINGO-1 increased the differentiation of neurons, but decreased the differentiation of astrocytes.

### Wnt5a protein expression is upregulated by LINGO-1 shRNA but not Wnt5a mRNA

**Fig. 5:**
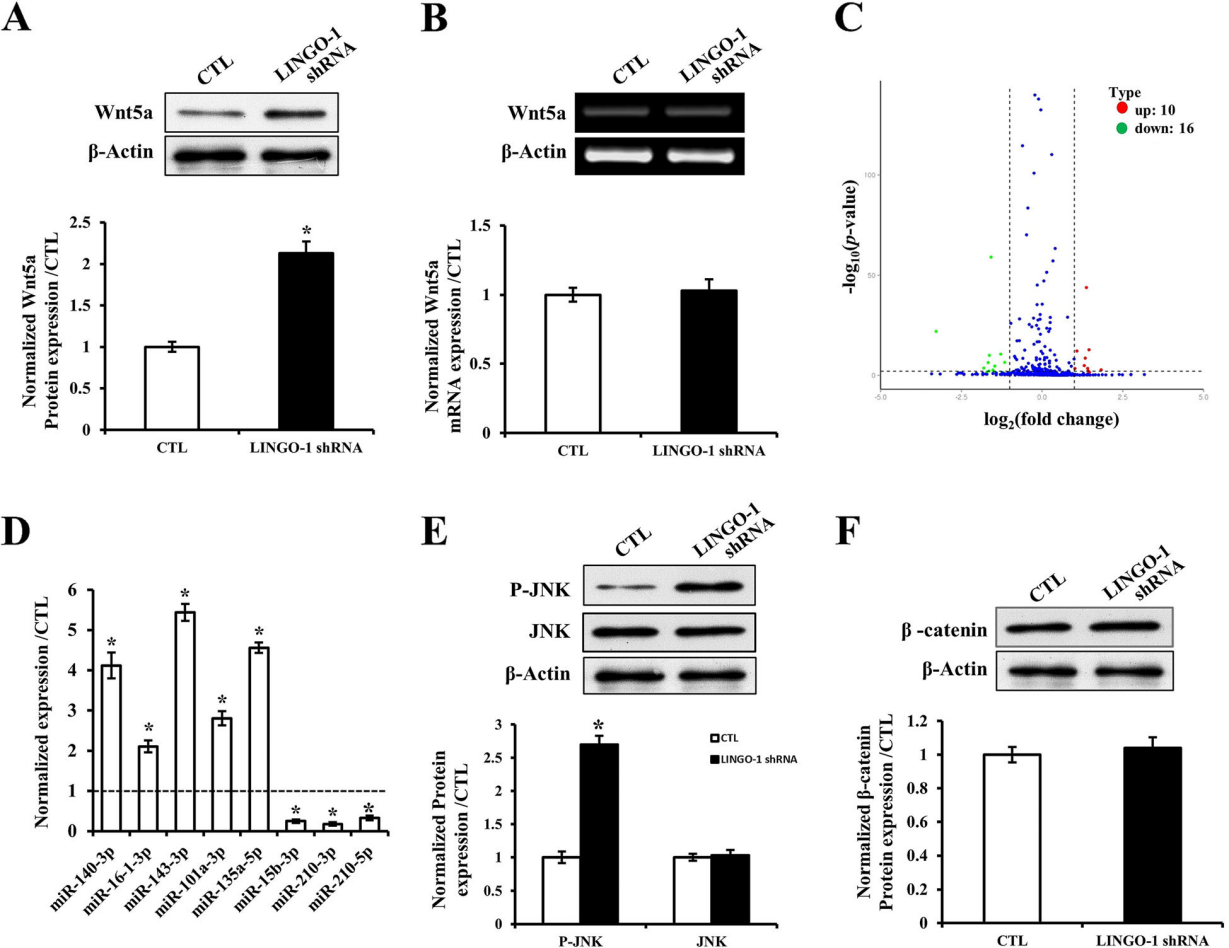
miR-15b-3p is downregulated in LINGO-1 shRNA-treated sp-NSPCs, resulting in increased expression of Wnt5a. **A** Wnt5a protein levels increased after LINGO-1 shRNA treatment. However, **B** its mRNA levels did not change. **C** Volcano plot analysis of miRNA levels between LINGO-1 shRNA and control NSPC groups. **D** qPCR of miRNAs in the two sp-NSPC groups showing miR-15b-3p was downregulated after LINGO-1 RNA interference. **E** Levels of p-JNK, JNK, and **F** β-catenin in LINGO-1 shRNA-treated sp-NSPCs. ^*^P < 0.05. Results are expressed as the mean ± SD. All of the analysis was calculated based on three biological replicates

The Wnt5a gene plays a critical role during NSPC differentiation. To investigate whether LINGO-1 had any effects on Wnt5a, we studied Wnt5a expression after inhibition of LINGO-1. The results showed that Wnt5a protein expression levels in the LINGO-1 shRNA group were significantly higher than those in the control group (*P* < 0.05) (Fig. 5A). However, Wnt5a mRNA levels were not altered (Fig. 5B), suggesting that Wnt5a protein levels may be regulated by post-transcriptional mechanisms.

To investigate this assumption, we considered a classical means of post-transcriptional regulation of gene expression: inhibition of translation by miRNAs. Many miRNAs are altered following SCI, and the involvement of miRNAs in numerous processes after SCI has been reported. Hence, we performed miRNA-seq to compare miRNA levels in LINGO-1 shRNA NSPCs and control NSPCs. The results showed that twenty-six miRNAs were significantly different between the two groups (Fig. 5C): ten miRNAs were upregulated and sixteen miRNAs were downregulated, among which some miRNAs were reported to have a relationship with neuronal development. To confirm these results, we evaluated eight miRNAs that changed significantly (according to the miRNA-seq analysis) by miRNA-specific qPCR. The results showed that three of these miRNAs were downregulated (miR-15b-3p, miR210-3p, miR210-5p) and five were upregulated (miR-140-3p, miR-16-1-3p, miR-143-3p, miR-101a-3p, miR-135a-5p), findings which were consistent with the miRNA-seq data (Fig. 5D).

Wnt5a is a non-canonical WNT molecule which plays an important role during cell differentiation and proliferation [11]. To investigate the signaling pathway that is activated by Wnt5a, we measured JNK and phosphor-JNK protein expression levels, since they are key molecules in the non-canonical Wnt signaling pathway. The results showed that phosphor-JNK protein levels in the LINGO-1 shRNA group were significantly higher (almost 2.7-fold) than in the control group (*P* < 0.05) (Fig. 5E). In contrast, the expression of β-catenin protein did not differ (Fig. 5F). These results suggest that LINGO-1 shRNA probably upregulates Wnt5a protein expressions.

### LINGO-1 regulates Wnt5a by modulating miR-15b-3p, inhibiting neural differentiation

**Fig. 6:**
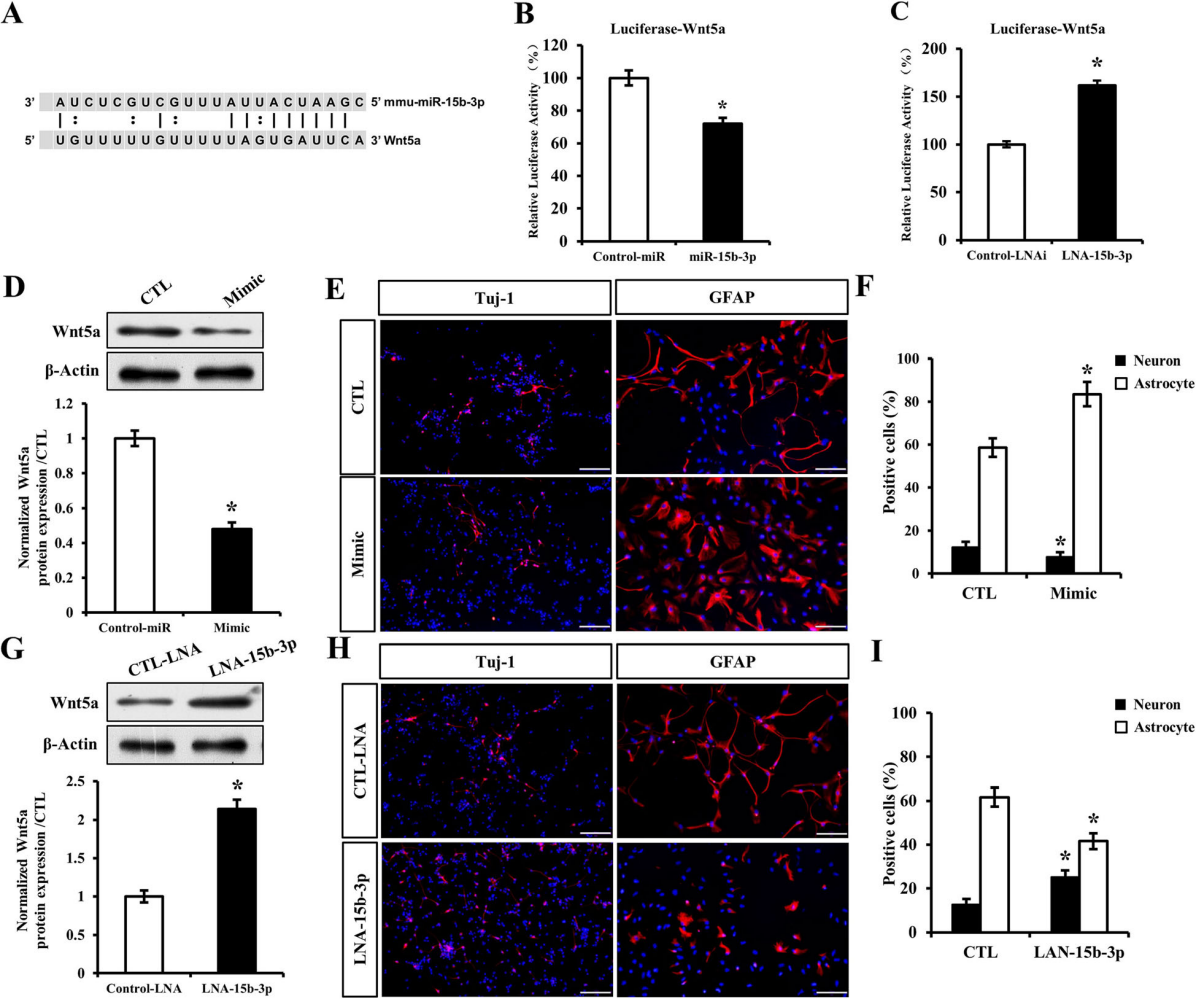
Wnt5a is the target of miR-15b-3p. **A** miR-15b-3p binding site in the 3′UTR of Wnt5a, as predicted by TargetScan. **B**, **C** Validation of miRNA binding to the 3′UTR of Wnt5a by luciferase activity assay. Reduction in luciferase activity demonstrated binding of miR-15-3p to the 3′UTR of Wnt5a. **D**–**F** Downregulation of Wnt5a protein levels by overexpression of miR-15b-3p in sp-NSPCs results in less neurons and more astrocytes, based on immunofluorescence staining and quantification. **G**–**I** Upregulation of Wnt5a protein levels after LNA inhibition of miR-15b-3p in sp-NSPCs results in more neurons but less astrocytes (bar = 100 μm), ^*^P < 0.05. Results are expressed as the mean ± SD. All of the analysis was calculated based on three biological replicates

Among eight miRNAs altered by LINGO-1 shRNA, miRNA-15b-3p was of particular interest, since the 3′ UTR of Wnt5a has one miRNA response element (MRE) for miR-15b-3p (Fig. 6A). To confirm the predicted binding of miR-15b-3p to the 3′ UTR of Wnt5a, we transiently expressed a miR-15b-3p mimic or an LNA inhibitor of miR-15b-3p in HEK293T cells together with luciferase reporter plasmids for Wnt5a 3′ UTR. The reduction and increase in luciferase activity in the presence of the miRNA mimic and LNA inhibitor, respectively, confirmed the presence of binding sites for miR-15b-3p in the 3′ UTR of Wnt5a (Fig. 6B, C).

To further confirm that miRNA-15b-3p influenced the expression of Wnt5a, leading to increased neural differentiation, we transiently expressed the miR-15b-3p mimic or LNA inhibitor of miR-15b-3p in NSPCs. As expected, endogenous Wnt5a protein expression levels were significantly downregulated in the presence of the miR-15b-3p mimic (Fig. 6D). Specifically, Wnt5a protein levels in the miR-15b-3p mimic group were only 47% of those in the control group. Furthermore, the percentage of Tuj-1-positive cells in the miR-15b-3p mimic group (7.8 ± 1.2%) was much lower than in the control group (12.3 ± 1.5%) (*P* < 0.05) (Fig. 6E, F). In contrast, Wnt5a protein expression was upregulated when miR-15b-3p was downregulated (Fig. 6G), and the percentage of Tuj-1-positive cells in the LAN-15b-3p group was significantly higher than in the control group (Fig. 6H). Specifically, the percentage of Tuj-1-positive cells in the LAN-15b-3p group (23.9 ± 1.8%) was almost 2-fold higher than that in the control group (9.8 ± 1.3%) (*P* < 0.05) (Fig. 6I).

### LINGO-1 downregulation influences sp-NSPC differentiation and promotes lesion recovery after SCI

**Fig. 7:**
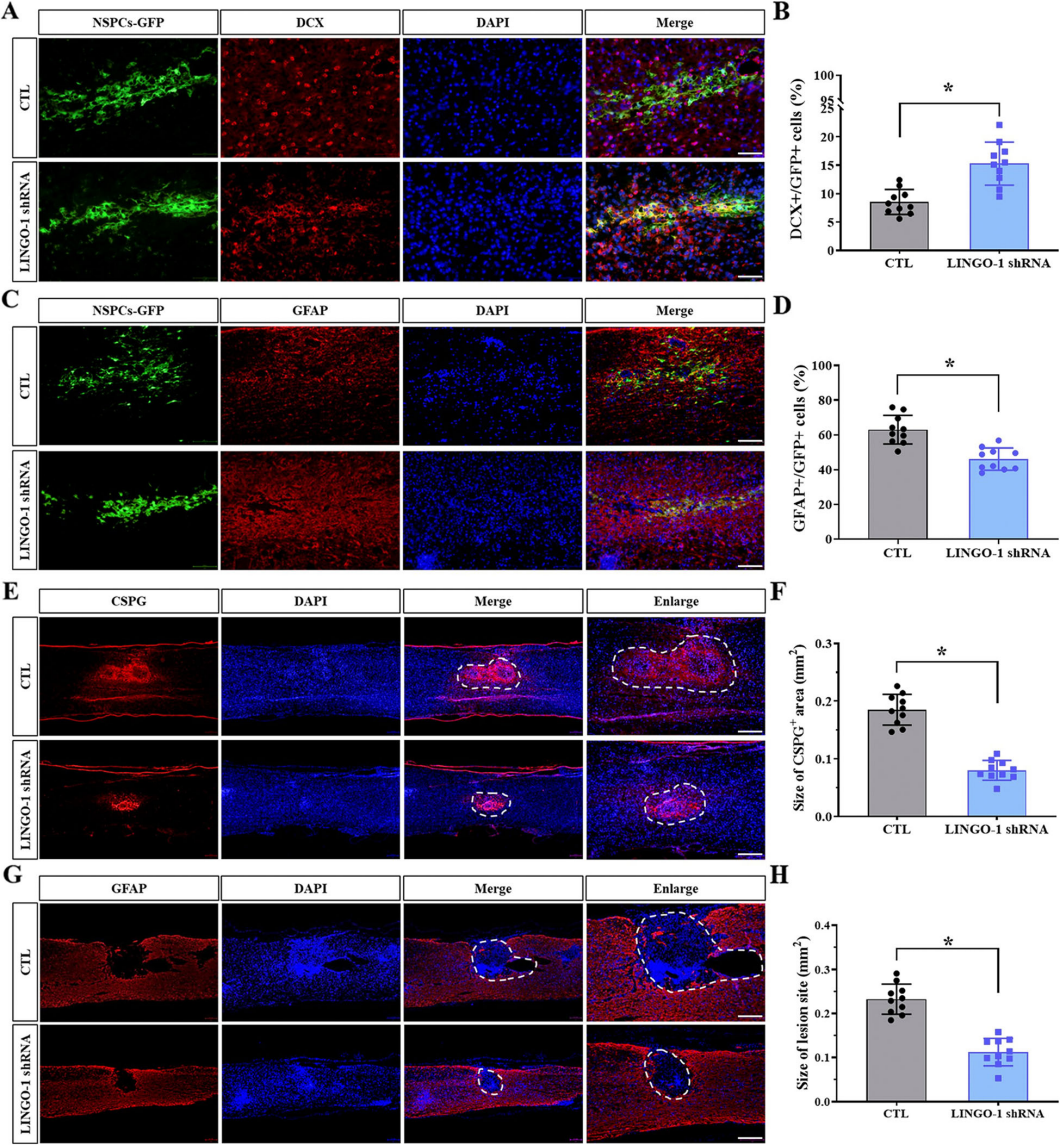
Transplantation of LINGO-1 shRNA-treated sp-NSPCs after SCI improves neuronal differentiation, has a cavity-filling effect, and promotes tissue repair. Immunofluorescent staining of sagittal sections of thoracic spinal cords at 35 d.p.i. showing **A**, **B** more differentiated neurons, as revealed by DCX staining; **C**, **D** less differentiated astrocytes, as revealed by GFAP staining; **E**, **F** reduced lesion volume, as revealed by CSPG staining; and **G**, **H** less cavities, as revealed by GFAP staining. n = 10 animals per group, unpaired two-tailed Student’s t-test. ^*^P < 0.05. Results are expressed as the mean ± SD

To investigate the function of LINGO-1 in sp-NSPCs injected into SCI mice, we first induced a moderate contusion at the T10 level that would result in spontaneous motor and sensory recovery over the course of 5 weeks. Following laminectomy at T9–10, contusion was induced by dropping a 5-g rod 6.25 mm above the spinal cord. Since LINGO-1 expression is continuously upregulated in vitro in mouse spinal cord-derived NSPCs and influences their differentiation, we tested whether downregulation of LINGO-1 would alter the differentiation of spinal cord-derived NSPCs in vivo. In agreement with the in vitro results, a 1.79-fold increase in DCX-positive cells, indicative of neuronal differentiation, was detected by immunofluorescence staining of the injured spinal cord (15.01 ± 3.60 vs 8.44 ± 2.13) (*P* < 0.05) (Fig. 7A, B). On the contrary, the percentage of GFAP-positive astrocytes in the LINGO-1 shRNA group (45.25 ± 6.23%) was much lower than that in the control group (62.45 ± 8.13%) (*P* < 0.05) (Fig. 7C, D).

Strikingly, the lesion size appeared to be much larger in the control group, with diffuse deposition of CSPGs detected by anti-chondroitin sulfate antibody. CSPGs are a family of matrix proteins which influence axon growth (*P* < 0.05, Fig. 7E, F). The size of the CSPGs+ area in the control group (0.185 ± 0.027 mm^2^) was 2.3 times larger than that in the LINGO-1 shRNA group (0.080 ± 0.017 mm^2^). Similar to the CSPG deposition findings, the lesion size in the control group based on GFAP staining (0.233 ± 0.027 mm^2^) was also larger than that in the LINGO-1 shRNA group (0.112 ± 0.031 mm^2^) (*P* < 0.05) (Fig. 7G, H). Tissue cavitation was observed occasionally at the lesion site in the control group only, although the central canal appeared enlarged in some sections from both groups owing to tissue distortion.

### Transplantation of sp-NSPCs with downregulated LINGO-1 promotes motor recovery after SCI

**Fig. 8:**
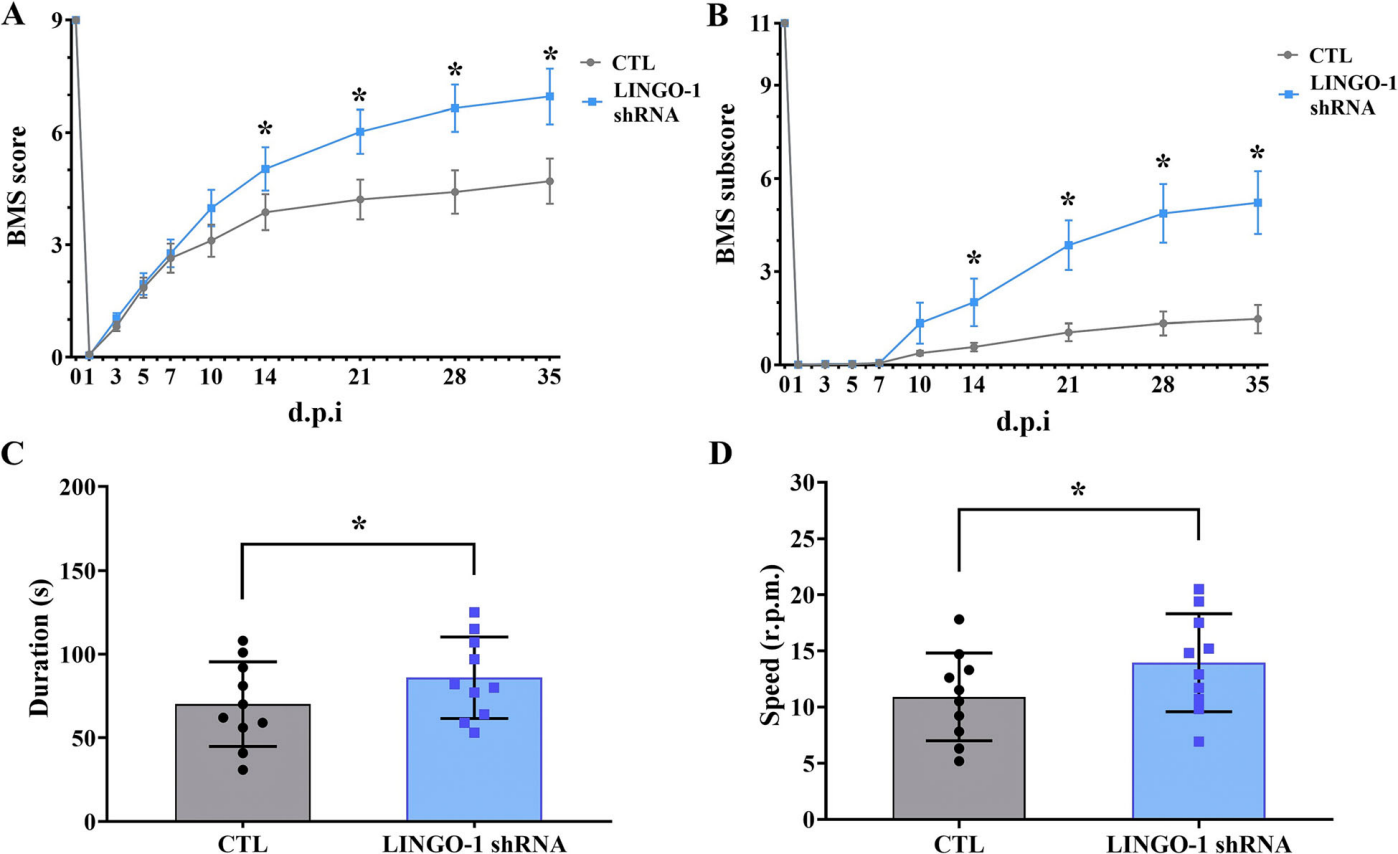
LINGO-1 downregulation is required for functional recovery after SCI. Time course of locomotor recovery in the LINGO-1 shRNA and control groups evaluated by means of **A** the 9-point Basso Mouse Scale (BMS) and **B** the 11-point BMS subscore. Motor function measured with the rotarod test (**C** duration and **D** speed) at 35 d.p.i. n = 10 animals per group, repeated measures analysis of variance (rmANOVA) with Bonferroni post hoc correction for BMS and the BMS subscore, unpaired two-tailed Student’s t-test for the rotarod test. ^*^P < 0.05. Results are expressed as the mean ± SD

Since LINGO-1 downregulation in sp-NSPCs promotes neuronal differentiation and reduces lesion size, we next examined whether transplantation of sp-NSPCs with downregulated LINGO-1 would facilitate functional recovery following SCI. Mice were subjected to spinal cord contusions and, 3 days after injury, received 1 μL (1 × 10^5^ cells/1 μL PBS) of LINGO-1 shRNA or control NSPCs by microinjections 2 mm rostral and caudal to the lesion epicenter. Mice transplanted with LINGO-1 shRNA-sp-NSPCs showed significantly better motor recovery than the control group throughout the 5 weeks, as determined by BMS and the BMS subscore for locomotion. As shown in Fig. 8, the BMS scores for the LINGO-1 shRNA group (6.96 ± 0.74) were significantly higher than those in the control group (4.71 ± 0.61) 2 weeks after the SCI (*P* < 0.05, Fig. 8A). In addition, significant improvement was seen in the BMS subscore, which evaluates finer aspects of locomotor control. This subscore improved from an average of 1.47 in the control group to 5.22 in the LINGO-1 shRNA group at day 35 post-injury (Fig. 8B).

Rotarod testing for hindlimb and tail balance was also carried out to evaluate motor recovery. Similar to the BMS assessment findings, the LINGO-1 shRNA group showed significantly better motor rehabilitation than the control group at the end of 5 weeks (*P* < 0.05). Rotarod duration and speed in the LINGO-1 shRNA group (85.90 ± 24.40 and 13.94 ± 4.36, respectively) were significantly higher than those in the control group (70.10 ± 25.28 and 10.89 ± 3.91, respectively) (Fig. 8C, D). Collectively, these results indicate that transplantation of sp-NSPCs with downregulated LINGO-1 promotes functional recovery after SCI.

## Discussion

Central nervous system regeneration is still a difficult goal in patients with spinal cord injury. Although NSPC transplantation is a promising treatment, manipulation of NSPC differentiation is required to counteract the inhibitory effects of the inflammatory microenvironment after SCI. Our research demonstrated that negative regulation of neural differentiation by LINGO-1 is, at least, partly mediated by upregulation of miR-15b-3p and the consequent translational inhibition of Wnt5a. Transplantation of LINGO-1 shRNA-treated sp-NSPCs improved motor function in SCI mice, highlighting its potential as a treatment for this condition.

In order to improve our understanding of the fundamental roles of LINGO-1 during the process of NSPC differentiation, we first harvested NSPCs from the spinal cords of mice after SCI. This is in contrast with previous studies [12], since Frisén reported that spinal cord-derived NSPCs have characteristic differences with respect to cortical and SVZ-derived NSPCs [13, 14]. In our experiments, LINGO-1 mRNA expression increased as the differentiation period progressed, whereas protein expression levels were only increased for the first few days, indicating that LINGO-1 may participate at the very beginning of this process, when sp-NSPC differentiation fate is determined. We then examined LINGO-1 expression in differentiated cells, such as neurons, astrocytes, and oligodendrocytes. Our results demonstrated that LINGO-1 was expressed in neurons and oligodendrocytes, but not in astrocytes. Lööv et al. reported similar results using cortical NSPCs [15]. We examined LINGO-1 expression in astrocytes on the 14th day of differentiation, whereas Lööv did so on the 6th day. The reason why LINGO-1 is not expressed in astrocytes is that LINGO-1 may influence the fate of astrocytes at the very beginning of differentiation, but not be active during the process of maturation. This would explain the LINGO-1 protein expression pattern in the Western blot analysis.

To investigate the influence of LINGO-1 on sp-NSPC differentiation, we used LINGO-1 shRNA to downregulate its expression. This was confirmed by qRT-PCR and Western blot analyses. We demonstrated that downregulation of LINGO-1 during sp-NSPC differentiation resulted in a 2-fold increase of Tuj-1-positive cells with respect to the control group, which is in agreement with previous reports [12, 15, 16]. Regarding the differentiation of astrocytes, there is still some controversy. We found a dramatic decrease in GFAP-positive cells after LINGO-1 shRNA treatment, but Lööv et al. found only a modest increase in the percentage of GFAP-positive cells in cultures where LINGO-1 was neutralized with antibodies. One probable reason for the distinct results is that we used RNA interference to downregulate LINGO-1 expression, which is the method used in other reports [12], whereas the other authors used the antibody neutralization method. Interestingly, although the percentage of GFAP-positive cells decreased, differentiated astrocytes showed a more ramified morphology. Since, according to our experiments, LINGO-1 was not expressed by astrocytes, it is possible that downstream cytokines could be promoting astrocyte maturation.

There are four main signaling pathways involved in the regulation of NSPC differentiation: bHLH [17], BMP [18], Notch [19], and Wnt [20]. Wnt signaling plays a key role in controlling stem cell fate [21]. This pathway has been divided into a canonical Wnt signaling pathway (GSK-3/β-catenin) involved in cell fate determination and non-canonical Wnt pathways (Ca^2+^ pathway and RhoA GTPase/cytoskeleton pathway) involved in the control of cell movement and tissue polarity. Wnt5a is a core factor in the non-canonical Wnt pathway and is a crucial neurogenesis regulator [22]. Jang et al. discovered that Wnt5a could activate neural differentiation by activating the Wnt5a/JNK pathway [23]. Li et al. also found that Wnt5a could promote NSC differentiation into neurons and further revealed that Wnt5a upregulated miRNA200b-3p expression through MAPK/JNK signaling, which was in accordance with our study [24]. In our study, we demonstrated that the expression of Wnt5a and p-JNK increased significantly during the differentiation of sp-NSPCs after LINGO-1 shRNA treatment, whereas the canonical Wnt pathway factor β-catenin did not change. More interestingly, although the expression of the Wnt5a protein increased significantly, its corresponding mRNA levels did not change, indicating that Wnt5a protein levels may be regulated by post-transcriptional mechanisms. This was indeed the case, as shown by miRNA sequencing. We discovered that a series of miRNAs changed after LINGO-1 shRNA treatment. Of these, 16 miRNAs were downregulated and 10 miRNAs were upregulated. Since miRNAs can target mRNAs, resulting in translational repression, this prompted us to identify a specific miRNA which could negatively regulate Wnt5a expression. To the best of our knowledge, this is the first demonstration that miR-15b-3p can target Wnt5a mRNA, resulting in increased Wnt5a expression and consequent promotion of neuronal differentiation. Since endogenous adult spinal cord stem cells fail to promote functional recovery efficiently [25], LINGO-1 shRNA-treated sp-NSPCs could be a promising strategy.

There are several mechanisms which may explain why NSPC transplantation can promote functional recovery after SCI [26], including inflammation modulation, axon regeneration promotion, bioactive molecule secretion, and replacement of lost cells. Although brain cortex/SVZ-derived NSPCs have been transplanted after SCI [27, 28], our study is the first to investigate the transplantation of LINGO-1 shRNA-sp-NSPCs as a way of promoting functional recovery. These cells can be obtained endogenously, especially after SCI. We observed a significant increase in neuronal differentiation and a reciprocal reduction in astrocyte differentiation, and this has been demonstrated in other studies. Interestingly, the proportion of differentiated neuronal cells was slightly lower in vivo than in vitro. This is probably due to the fact that the internal microenvironment is more complex, and other factors may negatively affect the differentiation of sp-NSPCs. On the other hand, the BMS score and BMS subscore increased substantially from day 14 onwards, reflecting an improved functional recovery in the LINGO-1 shRNA group, and supporting the potential use of LINGO-1 shRNA-sp-NSPCs to treat SCI. Similar results were observed in the rotarod test. The different outcomes between the two groups began to be detected on day 14, probably reflecting the maturation of differentiated sp-NSPCs. Above all, our study provides strong evidence that transplantation of LINGO-1 shRNA-sp-NSPCs is a promising approach to treat SCI.

## Conclusion

This investigation demonstrated positive effects of LINGO-1 shRNA on sp-NSPC differentiation, inducing an expansion of neurons and a reduction of astrocytes. The possible mechanisms involve the downregulation of miR-15b-3p and translational upregulation of Wnt5a. However, more details may still need to be elucidated. Our results also indicate that transplantation of LINGO-1 shRNA-treated sp-NSPCs can enhance recovery of motor function after SCI, highlighting its potential as a treatment for this condition.

## Data Availability

All data generated or analyzed during this study are included in this article.

## Abbreviations

SCI: Spinal cord injury
NSPCs: Neural stem and progenitor cells
MAIFs: Myelin-associated inhibitory factors
NgR: Nogo receptor
LINGO-1: LRR and Ig domain-containing, Nogo receptor-interacting protein
RNA-seq: RNA sequencing
qRT-PCR: Quantitative real-time PCR
PVDF: Polyvinylidene fluoride
TBST: Tris-buffered saline plus Tween
JNK: c-Jun N-terminal kinase
BMS: Basso Mouse Scale
bHLH: Basic helix-loop-helix protein
BMP: Bone morphogenetic protein
SVZ: Subventricular zone
